# Supplementary material for: The immunoglobulin A isotype of the Arabian camel (Camelus dromedarius) preserves the dualistic structure of unconventional single-domain and canonical heavy chains
Source: Front Immunol. 2023 Dec 12;14:1289769. doi: 10.3389/fimmu.2023.1289769 (PMC10756906; doi:10.3389/fimmu.2023.1289769)
Supplement: Supplementary file 1 [file DataSheet_1.docx]

**Supplementary file#1: caption with references**

**Primary structure of unconventional IgA heavy chains in the dromedary.** 101 amino acid sequences of dromedary unconventional IgA heavy chain, deduced from their corresponding cDNA templates originating from the nictitating membrane, were aligned according to IMGT^®^ delimitations using the MOE software (1). Amino acid residues are marked with different colors applying the UGENE software (2). The *bona fide* immunoglobulin fold of the variable domain consists of 9 antiparallel β strands (A, B, C, C’, C’’, D, E, F, G), which include four framework regions (FR1, FR2, FR3 and FR4), and three adjacent loops, *i.e.* BC-loop, C’C’’-loop, and FG-loop, that correspond to complementarity determining regions CDR1, CDR2 and CDR3, respectively. This distinctive structure is preserved in the variable domain of dromedary heavy chain-only IgA, termed the variable heavy heavy (V_H_H) domain. A conserved 19-mer leader peptide, MELGMSWVVLAALLQGVQA (MELG leader peptide), precedes the V_H_H domain, consisting of FR1, followed by CDR1, FR2, CDR2, FR3, CDR3 and FR4, which was separated from the C_H_α2 domain by a short, proline-rich hinge region (VPPPPPP). The V_H_H domains derive from a set of dromedary *IGHV3* genes (equivalent to human *IGHV3* family clan III). In our library, the highest frequency of *IGHV3* genes in the V-D-J rearranged CDR3 was found for *IGHV3-23*04* and *IGHV3-66*02*, followed by *IGHV3-74*02*. The multi-colored, mosaic-like appearance of CDR3, combined with length differences ranging from 14 to 26 amino acids (each dash indicates the absence of a residue), mirrors the typical hypervariability of antigen contact sites. C-terminal to CDR3, starting with FR4, followed by the proline-rich hinge region, constant domains C_H_α2 and C_H_α3, and ending with the invariant 18-amino-acid tailpiece, the variability of amino acid sequences abruptly ceases, marking the boundary between variable and constant domains. FR4 encompasses the C-terminal part of the *J* gene segment, with the canonical J motif W-G-X-G. In the V_H_H domain, unique, camelid-specific amino acid substitutions (phenylalanine for valine, glutamic acid for glycine, arginine for leucine, glycine for tryptophan) are found in FR2 (C strand and C’ strand), and serine replaced leucine in FR1 (A strand). Beside the exceptional length variability of CDR3 and the absence of C_H_α1, these substitutions represent the fingerprint of the V_H_H domain in heavy chain-only antibodies. The camelid-specific amino acid changes from hydrophobic to hydrophilic residues are positioned in the GFCC’C’’ β sheet of the immunoglobulin fold, which in the classic tetrameric configuration forms the V_H_-V_L_ interface, superfluous in the heavy chain-only variant. Apart from two invariant cysteines in FR1 (B strand) and FR3 (F strand), additional cysteines in CDR1 and CDR3 stabilize the conformation of the paratope in heavy chain-only antibodies. Of note is the unique amino acid composition of the 5’ end of the C_H_α3 domain, consisting of two anionic residues (E and D) in most of the IgA heavy chain-only sequences, except for sequence No 109 and No 111, where basic residues (R and H) were observed instead. The polar character of the N-terminal region of the C_H_α3 domain may account for a hitherto unexplored protein-protein interaction.

1. Molecular Operating Environment (MOE), 2022.02 Chemical Computing Group ULC, 910-1010 Sherbrooke St. W., Montreal, QC H3A 2R7, Canada (2023)

2. Okonechnikov K, Golosova O, Fursov M; UGENE team. Unipro UGENE: a unified bioinformatics toolkit. Bioinformatics (2012) 28(8):1166-7. doi: 10.1093/bioinformatics/bts091
