# Supplementary figures and images for: The immunoglobulin A isotype of the Arabian camel (Camelus dromedarius) preserves the dualistic structure of unconventional single-domain and canonical heavy chains
Source: Front Immunol. 2023 Dec 12;14:1289769. doi: 10.3389/fimmu.2023.1289769 (PMC10756906; doi:10.3389/fimmu.2023.1289769)

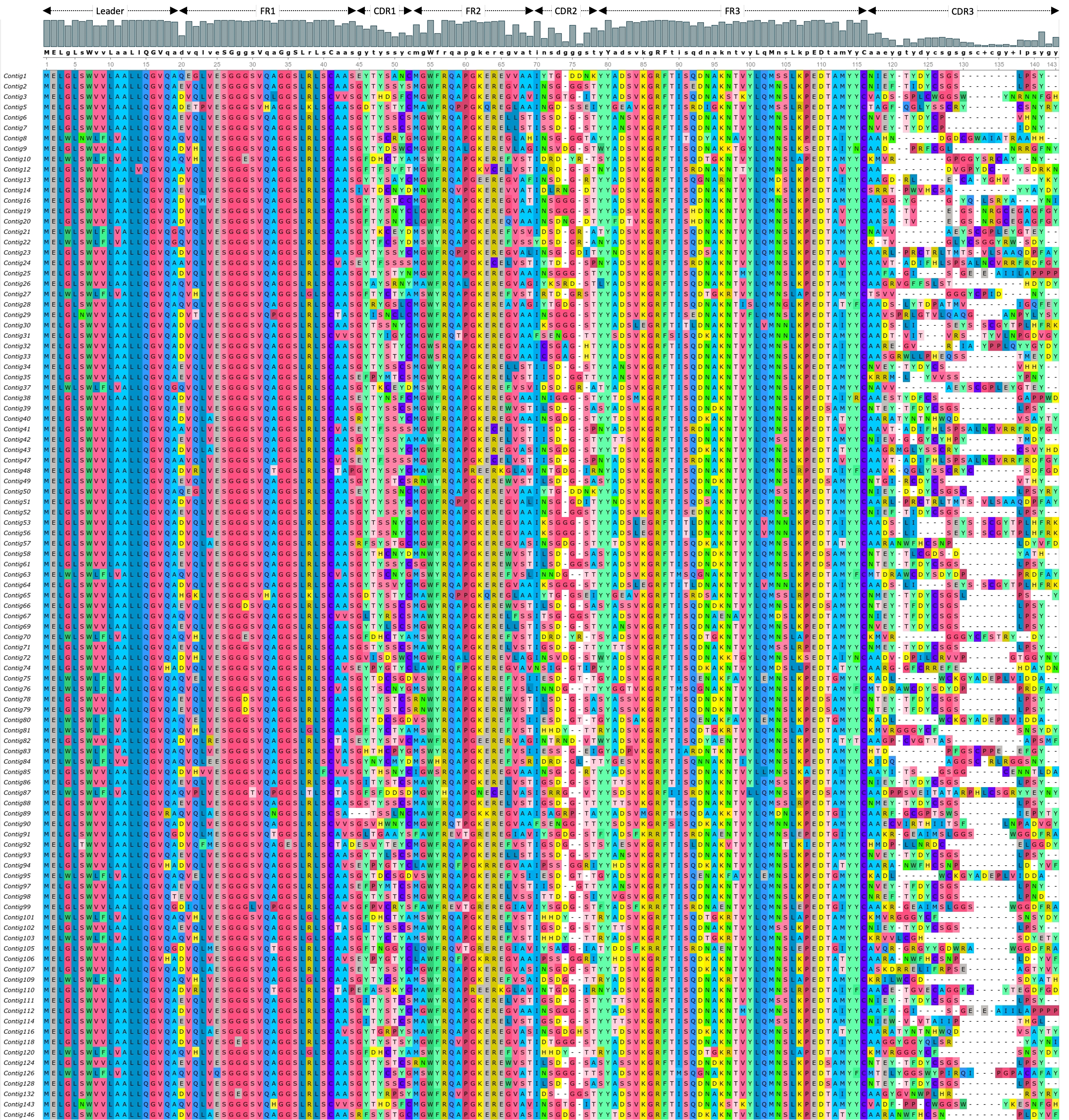

Supplement: Supplementary file 2 [file Image_1.tiff]

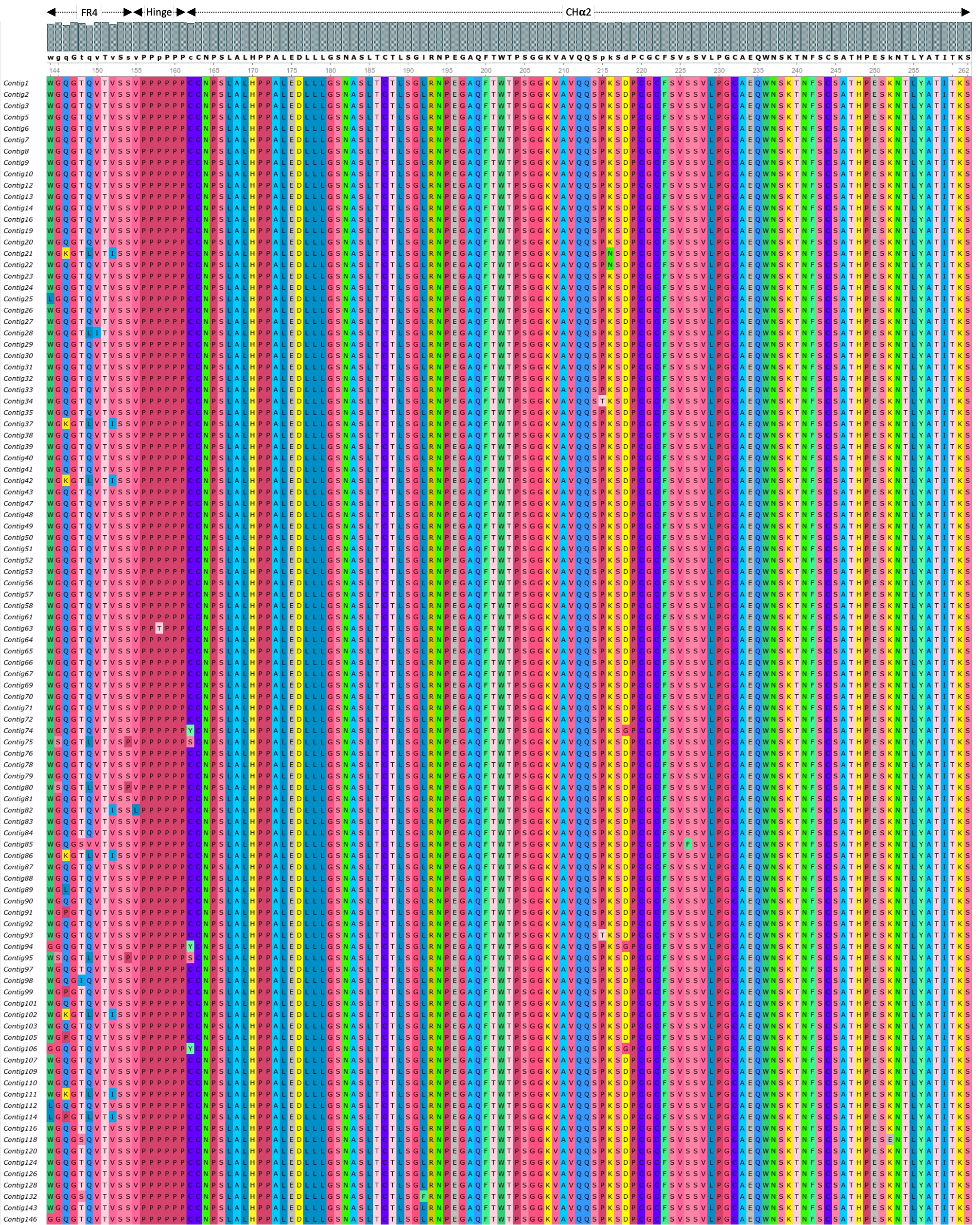

Supplement: Supplementary file 3 [file Image_2.tiff]

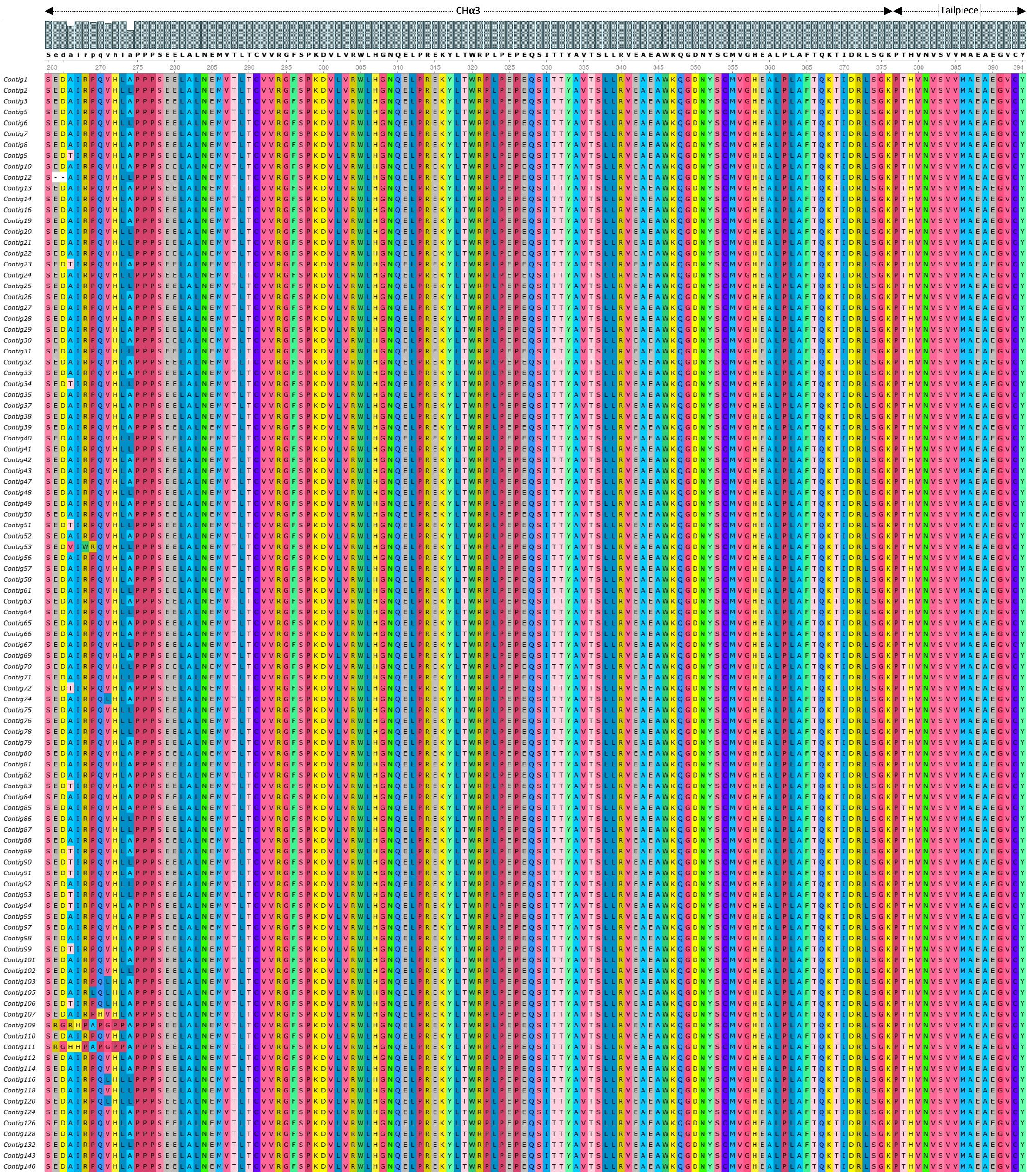

Supplement: Supplementary file 4 [file Image_3.tiff]
